# Snake bite mimicking brain death

**DOI:** 10.1186/1757-1626-1-16

**Published:** 2008-06-12

**Authors:** Joseph John, Bahubali D Gane, Nishad Plakkal, Rajeswari Aghoram, Sowmya Sampath

**Affiliations:** 1 Department of Pediatrics, Pondicherry Institute of Medical Sciences, Kalapet, Pondicherry 605 014, India; 2 Department of Medicine, Jawaharlal Institute of Postgraduate Medical Education and Research, Dhanvantri Nagar, Pondicherry 605 006, India

## Abstract

A 6 year old girl woke up with pain and increasing swelling over the left hand and difficulty in breathing. On examination, she had swelling of the left forearm and hand, flaccid quadriparesis and was in respiratory failure requiring mechanical ventilation. Two clean puncture wounds were identified on the left thumb. A provisional diagnosis of snake bite with severe envenomation was made and she was given anti snake venom therapy. Over a period of about 4 hours her weakness progressed. She became areflexic, developed internal and external ophthalmoplegia and loss of other brain stem reflexes mimicking brain death. Mechanical ventilation was continued despite features suggestive of brain stem dysfunction. About 36 hours after ventilation she showed a flicker of movement of her fingers and gradually the power improved. She was weaned off the ventilator and extubated after 5 days. External ophthalmoplegia is an established association with cobra envenomation, but, this combination of internal and external ophthalmoplegia can mimic brain death and pose a dilemma to the caregivers regarding continuation of therapy.

## Case report

A 6 year old girl who was sleeping on the floor woke up at 3 AM with pain and swelling over the left hand. This was initially ignored by the parents. The pain and swelling gradually increased, and she complained of difficulty in breathing about 2 hours later. At this point she was brought to the hospital. There was no history suggestive of a snake bite. Examination revealed a drowsy child with a massively swollen left hand and forearm with shallow breathing. There were two clean puncture marks on the left thumb but no bleeding from them. There was no blistering or necrosis of the swollen limb. The local lymph nodes were enlarged. Examination of the central nervous system showed the child to be drowsy but arousable, not obeying verbal commands and was localizing pain. She had ptosis and grade 2–3/5 power in all four limbs and sluggish deep tendon reflexes with extensor plantars. Her pulse rate was 80/min, BP-100/60 mmHg SpO_2 _– 60% on 5 liters of oxygen by face mask and temperature 98.4°F. Endotracheal intubation was done and she was ventilated on synchronized intermittent mandatory ventilation (SIMV) mode as she had some spontaneous respiratory efforts. Polyvalent anti snake venom therapy was started and a total of 100 ml was given (Haffkine Institute, Bombay), after a provisional diagnosis of snake bite with severe envenomation was made. Over a period of about 4 hours, the weakness progressed and involved proximal muscles first and then distal muscles. She was comatose, had no motor response to painful stimuli, became areflexic and her plantar reflex was not elicitable. Her pupils were dilated with no response to light; she had absent oculocephalic and corneal reflexes. She had no spontaneous respiratory effort, no response on tracheal suctioning and went onto full ventilatory support. Ventilation was continued despite findings suggestive of brain stem dysfunction and after about 36 hours of ventilation she showed a flicker of movement of her fingers and toes. The paralysis improved distally first then proximally. She was weaned off the ventilator and extubated after 5 days of ventilation. From about 36 hours of ventilation she had been noticed to have high blood pressure recordings up to 160/100 mm Hg which over a period of three days returned to normal without any specific drug therapy. When she was shifted out of the PICU she had grade 3 power in the lower limbs grade 4 power in the upper limbs and was breathing well but had truncal muscle weakness. She had mid-dilated and very sluggishly reacting pupils. She had no dysphagia or dysphonia. At discharge from hospital she had truncal muscle weakness, grade 4 power in the lower limbs, grade 5 power in the upper limbs, her pupils were still mid dilated and reacting sluggishly to light. The local reaction in the left upper limb had settled.

## Discussion

Venomous snakes are classified into two important families, elapidae and viperidae. Elapidae have short, permanently erect fangs and include cobra, krait, coral snakes and sea snakes. Viperidae on the other hand have long fangs folded up against the upper jaw which are erected when the snake strikes. This family consists of snakes like the typical vipers (Viperinae) and the pit vipers (Crotalinae). The Crotalinae have a special sense organ called the pit organ situated between the nostril and the eye to detect warm blooded prey.[1]

There are no reliable reports on the incidence of snake bites in India as many snake bite victims are treated by traditional practitioners and not in hospitals. An estimated 35000–50000 people die of snake bite each year in India.[1] Snake bites are more common after rains, after floods, during harvest and at night.[1,2] Many bites like the one in our case occur at night when the snake enters the house in search of its prey and people sleeping on the floor may be bitten. In many cases the history of a snake bite may not be forthcoming (as in the present case).

Neuromuscular paralysis occurs due to blockade of neuromuscular transmission. Cobra venom acts post synaptically while krait venom acts pre synaptically.[1] Polypeptide neurotoxins cause muscle paralysis by blocking the nicotinic acetylcholine receptors at the post-synaptic motor endplates, or they affect the mode of neurotransmitter release at the presynaptic motor nerve endings.[3] Neurotoxic paralysis may also begin within the first hour with ptosis being one of the earliest manifestations, followed by external ophthalmoplegia.[1,3] The binding to the presynaptic portion is irreversible, hence clinical recovery occurs slowly and only with the formation of a new neuromuscular junctions. The binding of toxin to the postsynaptic portion produces a competitive or noncompetitive acetylcholine receptor blockade.[3] Although an antivenom may induce a certain degree of reversal of the paralysis by postsynaptic neurotoxin, the clinical recovery is, however, very slow. Paralysis involves the proximal muscles first and then the distal muscles and recovery occurs in the reverse order.[1] The internal ophthalmoplegia is attributed to autonomic dysfunction.[4]

This case highlights the occurrence of both internal and external ophthalmoplegia which would mimic brain death in many ways, thus prompting many a pediatrician to consider withdrawing ventilatory support, which would be disastrous. In such a case other confirmatory tests of brain death like electroencephalography, four vessel cerebral angiography, Transcranial Doppler ultrasonography or radionuclide imaging (Technetium Tc 99 m hexametazime) should be resorted to.[5] Supportive care needs to be continued till the effects of the venom wear off with excellent outcomes.

## Consent

Written informed consent was obtained from the patient for publication of this case report. A copy of the written consent is available for review by the Editor-in-Chief of this journal

## Competing interests

The authors declare that they have no competing interests.

## Contributions by Authors

Dr JJ was involved in the case management, drafted the manuscript and will act as the guarantor, Drs BDG and NP were involved in the case management reviewed the draft manuscript and suggested revisions, Dr RA and SS reviewed the manuscript and made the final corrections before submission

## References

[B1] Warrell DA, international panel of experts (1999). Guidelines for the Clinical Management of Snake Bite in the South-East Asia Region. Southeast Asian J Trop Med Public Health.

[B2] Sharma N, Chauhan S, Faruqi S (2005). Snake envenomation in a north Indian hospital. Emerg Med J.

[B3] Lee Sung Woo, Jung In Chul, Yoon Young Hoon (2004). Anticholinesterase Therapy for Patients with Ophthalmoplegia Following Snake Bites: Report of Two Cases. J Korean Med Sci.

[B4] Agarwal R, Singh N, Gupta D (2006). Is the patient brain dead?. Emerg Med J.

[B5] Eelco FM, Wijdicks N (2001). The diagnosis of brain death. N Engl J Med.

